# Healthcare Priorities for Surgical Care in Canada

**DOI:** 10.1097/AS9.0000000000000561

**Published:** 2025-04-04

**Authors:** Kaitlyn Squires, Mercedes Pilkington, Louise Clément, Mary E. Brindle

**Affiliations:** From the * University of Calgary, EQuIS Research Platform, Calgary, Alberta, Canada; † Hospital for Sick Children, Pediatric General and Thoracic Surgery, Toronto, Ontario, Canada; ‡ Ariadne Labs, Brigham and Women’s Hospital, Harvard T.H. Chan School of Public Health, Boston, MA; § Health Standards Organization/Accreditation Canada, Ottawa, Ontario, Canada; ‖ Alberta Children’s Hospital, Pediatric General Surgery, Calgary, Alberta, Canada.

**Keywords:** surgical safety, quality, Canada, population

## Abstract

Surgical disease is one of the most costly and morbid areas of health care and, perhaps, the most amenable to improvement through well-designed and well-implemented tools. Preventable adverse events have been successfully targeted by tools such as the Surgical Safety Checklist. However, health system priorities related to surgery have evolved; the concepts of what constitutes quality care have changed over time. How efficiency and access contribute to safe care, where equity exists as a key outcome measure, and the central role of the patient in defining good care have changed the way we look at surgical priorities. This review of Canadian surgical priorities provides a unique perspective on how we should consider national strategies for surgical safety by exploring both nationally published data as well as the priorities set by provincial and governmental agencies that extend beyond traditional clinical metrics.

## INTRODUCTION

Surgical care is one of the costliest sectors impacting national health systems and is associated with a significant burden of patient morbidity. Nearly 2 decades ago, the Canadian Adverse Events Study demonstrated that just over half of adverse events affecting hospitalized patients impacted those admitted to a surgical service.^1^ Among patients admitted to acute care hospitals in Canada, a disappointing, but perhaps not surprising, 7.5% were found to experience one or more adverse events; of which, 36.9% were deemed highly preventable.^1^ This study, alongside the Harvard Medical Practice Study in the United States and the Institute of Medicine’s To Err is Human report,^2–4^ served as an urgent appeal to improve the safety of inpatient surgical care. This call to action has been taken up globally as outlined in the Global Patient Safety Action Plan by the World Health Organization, which establishes priorities to eliminate avoidable harm and increase patient safety in healthcare.^5^ Despite the call for a global commitment to improved patient safety, the Canadian Institute for Health Information (CIHI) reported that for the 2022 to 2023 fiscal year, one in 17 inpatients in Canada experienced one or more adverse events.^6^ Although this is less than what was demonstrated nearly 20 years ago, it is an increase of 13% since the 2014 to 2015 fiscal year.^6^ Progress initially made, now appears to be lost. Patients undergoing surgery still have the highest rate of safety incidents of all patients admitted to hospital and the highest rates of complex adverse events.^7,8^ Despite the increasing recognition of the role of healthcare delivery in causing harm, there remains a fragmented assessment and approach to surgical safety.^9–11^ The attributable cost of healthcare harm is ballooning,^12^ and it is imperative to understand contemporary surgical safety priorities, national benchmarks, and the existing methodologies used to target and reduce perioperative safety events. At the same time, there are opportunities to improve by learning from the examples set by resilient and successful health systems.

Surgical care must be made safer for Canadian patients; however, the priorities we set to achieve that goal vary. To accomplish a system-wide change, prioritization of targets for surgical care improvement and the establishment of indicators by which to measure progress is required. Over the years, this exercise has been undertaken separately by a number of governmental organizations in Canada. Canada has a unique system of 13 separate provincial and territorial healthcare systems that are responsible for the organization and delivery of healthcare to their residents, and a federal health system that, among other responsibilities, provides funds to the provinces and establishes national standards. To inform a unified Canadian strategy for surgical safety, we have performed a narrative review within and across these provincial and national organizations to identify unique regional priorities and inform national alignment and investment.

## METHODS

For this review, we focused not on peer-reviewed publications but, rather, on priorities outlined by national and provincial organizations and published on organization websites, publicly available government documents, or as position statements/white pages in journals. We identified these organizations through federal and provincial websites and identification by health system leaders, with supplementation by electronic searches performed by two separate investigators and cross-referenced to ensure completeness. We prioritized national and provincial government/decision-making bodies and explored published surgical safety priorities from professional societies.

We reviewed the contemporary priorities of these Canadian national and provincial organizations specifically as they relate to surgical safety. There are a number of organizations in Canada that have targeted healthcare safety as a priority. One of the most notable collaborations was the National Patient Safety Consortium, which brought together Canadian safety leaders to define patient safety from 2014 to 2016.^7^ This process was convened by the former Canadian Patient Safety Institute (now Healthcare Excellence Canada) and CIHI. Although the consortium was only a temporary collaborative body, there are numerous contemporary organizations that have established surgical safety priorities in Canada. Fifty-six organizations were included in the review, representing both national and provincial healthcare priorities, including federal and provincial health authorities and Ministries of Health, patient safety and quality councils, research and quality improvement platforms, healthcare accreditation bodies, public health organizations, medicolegal and insurance associations, and healthcare professional associations (Table 1).

**TABLE 1.** Canadian Healthcare Organizations With Identified Surgical Priorities

Organization	Description	Organization	Description
Accreditation Canada	National healthcare accreditation body	Newfoundland & Labrador Department of Health and Community Services	Provincial Ministry of Health
Alberta Health Services	Provincial health authority	NL Health Services	Provincial health authority
Alberta Ministry of Health	Provincial Ministry of Health	Northwest Territories Department of Health and Social Services	Provincial Ministry of Health
British Columbia (BC) Centre for Disease Control	Provincial authority public health program	Nova Scotia Department of Health and Wellness	Provincial Ministry of Health
BC Provincial Health Services Authority	Provincial health authority	Nova Scotia Health	Provincial health authority
BC Ministry of Health	Provincial Ministry of Health	Ontario Ministry of Health	Provincial Ministry of Health
Health Quality BC	Provincial quality improvement agency	Operating Room Nurses Association of Canada	Healthcare professional organization
Best Practices in Surgery	University-led quality improvement organization	Patients for Patient Safety Canada	National quality improvement agency program
Canadian Anesthesiologists’ Society	Healthcare professional organization	PEI Department of Health and Wellness	Provincial Ministry of Health
Canadian Association of General Surgeons	Healthcare professional organization	Royal College of Physicians and Surgeons of Canada	National organization for physician standards
Canadian Association of Thoracic Surgeons	Healthcare professional organization	Quebec Ministry of Health and Social Services	Provincial Ministry of Health
Canadian Institute for Health Information	Independent, nonprofit healthcare organization	Saskatchewan Health Authority	Provincial health authority
Canadian Medical Association	Healthcare professional organization	Saskatchewan Ministry of Health	Provincial Ministry of Health
Canadian Medical Protective Association	Mutual medical defence organization	Shared Health Manitoba	Provincial health authority
Canadian Neurosurgical Society	Healthcare professional organization	Vitalité Health Network (NB)	Provincial health authority
Canadian Nosocomial Infection Surveillance Program	Provincial authority public health program	Yukon Health and Social Services	Provincial Ministry of Health
Canadian Nurses Association	Healthcare professional organization	Newfoundland & Labrador Department of Health and Community Services	Provincial Ministry of Health
Canadian Orthopedic Association	Healthcare professional organization	NL Health Services	Provincial health authority
Canadian Society for Vascular Surgery	Healthcare professional organization	Northwest Territories Department of Health and Social Services	Provincial Ministry of Health
Canadian Society of Colorectal Surgeons	Healthcare professional organization	Nova Scotia Department of Health and Wellness	Provincial Ministry of Health
Canadian Society of Endoscopic Surgery	Healthcare professional organization	Nova Scotia Health	Provincial health authority
Canadian Society of Hospital Pharmacists	Healthcare professional organization	Ontario Ministry of Health	Provincial Ministry of Health
Canadian Society of Pediatric Surgeons	Healthcare professional organization	Operating Room Nurses Association of Canada	Healthcare professional organization
Canadian Society of Plastic Surgeons	Healthcare professional organization		
Canadian Society of Respiratory Therapists	Healthcare professional organization		
Canadian Urological Association	National professional organization		
Canadian Vascular Society	National professional organization		
Enhanced Recovery Canada	National quality improvement agency program		
Health Canada	National health authority		
Health PEI	Provincial health authority		
Health Quality Council of Alberta	Provincial quality improvement agency		
Health Quality Ontario	Provincial quality improvement agency		
Healthcare Excellence Canada	National safety and quality improvement agency		
Healthcare Insurance Reciprocal of Canada	Healthcare organization insurance provider		
Horizon Health Network (NB)	Provincial health authority		
Infection Prevention & Control Canada	National, multidisciplinary non-profit organization		
Manitoba Health	Provincial Ministry of Health		
New Brunswick Department of Health	Provincial Ministry of Health		

To understand surgical safety priorities in Canada, we examined the current (or most recently available) strategic initiative documents of all included organizations. We performed our searches between November 2023 and March 2024. Priority areas outlined in the strategic documents were extracted and organized thematically into either surgery or healthcare (general). Each priority recorded by at least five organizations was deemed relevant for national priority setting and included in the results. The identified surgical priorities were utilized as the basis for a call to action and recommendations for priority setting to improve surgical safety.

## RESULTS

Surgical safety is a focus for a multitude of national stakeholders. A shared national mission for surgical safety in Canada has not been articulated but the elements that would contribute to such a mission are apparent when we look across the position statements and publications of national health organizations. The need for Canada to develop a surgical safety strategy has been identified by clinician leaders at the frontline of surgical care.^13^ There are eight surgical priorities that have been identified by at least five large-scale organizations (Table 2; Supplementary Material 1, see http://links.lww.com/AOSO/A483). The most frequently stated priorities were addressing surgical waitlists, improving patient safety, and enhancing quality of care.

**TABLE 2.** Identified Surgical Priorities With Representative Quotes From Canadian Healthcare Organizations

Surgical Priorities	Exemplar
1. Addressing surgical wait time and efficiencies	“Improve surgical wait times by: reviewing a centralized booking system to better manage waitlists and remove administrative inefficiencies, consistently implementing best practices to align with national benchmarks, [and] addressing delays resulting from the COVID-19 pandemic through allocation strategies based on patient need”. Nova Scotia Department of Health and Wellness (p. 5)^45^
2. Improving patient safety and quality of care	“Providing [surgical] care which is safe and of high-quality … [and is] clinically appropriate, evidence-based, and guided by standardized care pathways” (p. 18). Alberta Health Services (p. 18)^46^
3. Improving patient outcomes	“[The Enhanced Recovery Canada] evidence-informed principles support better outcomes for surgical patients including: an improved patient experience, reduced length of stay, decreased complication rates and fewer hospital readmissions.” Enhanced Recovery Canada (para. 6)^47^
4. Improving care coordination and surgical pathways (eg, handovers, transitions)	“Expand access to safe, high quality, coordinated health services, close to home for the people we serve by: enhancing team-based primary health care and improving health outcomes through health networks, ensuring continuity of care at every health encounter, [and] developing collaborative inpatient care models” Saskatchewan Health Authority (p. 7)^48^
5. Establishing perioperative service standards and best practices	“[Developing] quality standards in areas where there appears to be large variations in care or where there is a gap between the best possible care and the quality of care that is currently provided” Health Quality Ontario (p. 13)^49^
6. Improving surgical culture, teamwork, and communication	“Strengthening their collaborative skills helps to build healthy environments with strong communication and cohesive teamwork.” BC Patient Safety & Quality Council (p. 16)^50^
7. Reducing preventable harm and never events	“Providing care to patients is a complex endeavor, and risk is unavoidable. However, health organizations and workers have the knowledge and ability to reduce the occurrence of [never] events and should strive to prevent them entirely (eg, surgery on the wrong body part, the wrong patient, or the wrong procedure, or unintended foreign object left in a patient following a procedure)” Canadian Medical Protective Association (p. 13)^38^
8. Reducing surgical site infections	“Surgical site infection is the most common healthcare-associated infection among surgical patients, with 77 percent of patient deaths reported to be related to infection. [While] advances have been made in infection control practices, … [surgical site infection] remains a substantial cause of morbidity, prolonged hospitalization, and death” Canadian Patient Safety Institute (p. 4)^37^

Several commonalities can be identified across national, provincial, and territorial organizational priorities. The overall mandate for most organizations relates to the quality and safety across entire health systems of which surgical care is only a component. While many priorities are not surgery-specific, they interface with surgical patients and providers. This includes improving patient outcomes, patient experiences, and system safety; enhancing quality of care; and improving access to care. Patient-reported outcome measures and patient-reported experience measures are recognized as valuable, patient-centered tools for health system and service quality improvement.^14,15^ The prioritization of patient-centered approaches is reflected in the mission statements of organizations such as the Canadian Medical Association: “a health system that’s sustainable, more accessible and patient partnered” (p. 4)^16^ and provincial organizations such as the Alberta Quality Council: “to promote and improve patient safety, person-centred care, and health service quality throughout Alberta” (p. 8).^17^ Many of these organizations highlight surgical examples when emphasizing the importance of a patient-centered approach.^18^ Surgical professional organizations, likewise, promote patient-centered care in mission statements and priorities (eg, the Canadian Association of General Surgeons and the Canadian Orthopedic Association).

Perioperative professional organizations are well-positioned to identify patient populations at risk of disparate outcomes. For example, addressing the increasing waitlists for hip replacement has been identified as a national priority by the Canadian Orthopedic Association, providing a specific target population for national and provincial organizations aiming to improve access to care. Reducing surgical waitlists is a common organizational priority that goes hand-in-hand with improving health system capacity.^19^

Along with broad principle-base priorities, there are several specific and timely healthcare issues raised repeatedly by national organizations: pandemic recovery (increasingly replaced with the long-standing issue of surgical waitlists), mental health and addictions, long-term care, the opioid crisis, and antimicrobial resistance (Table 3; Supplemental Material 2, see http://links.lww.com/AOSO/A483). Many of these priorities have clear surgical connections. For example, postoperative pain and prescribing practices have been linked to opioid overuse and addictions,^20^ inappropriate antibiotics for prophylaxis and treatment of surgical conditions increase resistance,^21,22^ and surgical procedures frequently act as the catalyst for the loss of independence in older adults and the need for long-term care.^23,24^

**TABLE 3.** Identified Healthcare Priorities With Representative Quotes From Canadian Healthcare Organizations

Healthcare Priorities	Exemplar
Pandemic recovery	“Together, we can not only recover from the effects of the pandemic, but also build more resilient, equitable and innovative ways of designing, funding and delivering care in the future” Healthcare Excellence Canada (p. 8)^47^
Mental health and addictions	“Health Canada will focus on expanding the delivery of high-quality, accessible and free mental health and substance use services” Health Canada (p. 3)^51^
Long-term care	“Improve range of supports for people in long-term care homes to ensure they receive dignified and quality care, working with care providers, embedding person-centered respect and compassion in all service delivery” BC Ministry of Health (p. 10)^52^
Opioid crisis	“Improving access to harm reduction and treatment services, naloxone training and distribution, and the safer supply of prescription opioids” Health Canada (p. 31)^51^
Antimicrobial resistance	“Antimicrobial resistance [(AMR)] is known to impact length of stay and healthcare costs [and] it is expected that by 2050 an estimated 10 million annual deaths will be attributable to AMR; thus, antimicrobial susceptibility information is key to ensuring appropriate treatment and use of antimicrobials to help reduce AMR” Canadian Nosocomial Infection Surveillance Program (p. 326)^53^

Addressing issues of equity has been consistently identified in contemporary health system priorities. Although issues of equity in access are most often cited (and have been increasingly targeted with strategies to decrease surgical waitlists), other inequities in care have been identified by multiple organizations and include the provision of culturally safe care (ie, recognizing cultural and diverse needs of patients).

## SOLUTIONS TO ADDRESS CANADIAN SURGICAL SAFETY PRIORITIES

Several national health organizations including subspecialty societies have identified potential solutions that might address existing safety gaps and priorities for surgical care. These solutions have been outlined by numerous organizations and include integrating care, safe transitions, community-based care, and virtual care options (Table 4). The number of solutions identified is varied but some key areas have been commonly targeted by national and provincial organizations.

**TABLE 4.** Strategies to Address Surgical Priorities

Surgical Priorities	Strategies to Address Priorities
1. Addressing surgical backlogs and efficiencies	“To respond to patients’ needs, and to provide relief to those who have been waiting, we must increase capacity” through increasing surgeries, essential personnel, and access to diagnostic services; focusing on patients; adding more resources; and reporting on progress. BC Ministry of Health (p. 7)^54^
2. Improving patient safety and quality of care	“Change will be deliberate and focused, and will align to a holistic, values-based approach that ensures all dimensions of quality—acceptability, accessibility, appropriateness, efficiency, effectiveness, safety and equity—are considered when measuring success” Alberta Health Services (p. 7)^55^
3. Improving patient outcomes	The Ontario Surgical Quality Improvement Network, which “[brings] together surgical teams from all specialties and hospital types from across the province to form a community dedicated to quality improvement in surgery for better patient outcomes” Health Quality Ontario (p. 4)^39^
4. Improving care coordination and surgical pathways (eg, handovers, transitions)	“Enhanced recovery approaches also improve patient and healthcare provider experiences by helping clinicians and patients work as a coordinated team” Enhanced Recovery Canada (para. 3)^56^
5. Establishing perioperative service standards and best practices	“By embedding explicit expectations into our evidence-based standards and programs, we empower people to change their behaviour and practices to achieve better quality outcomes” Accreditation Canada (p. 9)^57^
6. Improving surgical culture, teamwork, and communication	“Supporting teams in building the capability to interact and work together effectively” BC Patient Safety & Quality Council (p. 7)^50^
7. Reducing preventable harm and never events (eg, foreign body retention, wrong-side/patient/procedure surgery)	CIHI developed a number of quality and safety indicators, such as the Hospital Harm indicator, which collects data on a wide range of potentially preventable harm. Indicators as such “help inform [hospital’s] quality initiatives and ultimately improve care for their patients” Canadian Institute for Health Information (para. 4)^58^
8. Reducing surgical site infections	“Establish ongoing national surveillance system to determine the epidemiology of nosocomial infections in Canadian hospitals; establish “benchmark” data; [and provide data that can be used to develop national guidelines for infection prevention” Infection Prevention and Control Canada (para. 10)^59^

## NATIONAL METRICS AND BENCHMARKS FOR SURGICAL SAFETY

To drive improvements in Canadian surgical performance, standardized, important, and actionable metrics are critical. A single adverse events study has been conducted in Canada.^1^ Despite the call for action that this study provided almost 20 years ago, there has yet to be a major change in the rates of adverse events occurring across the country.

**FIGURE 1.**
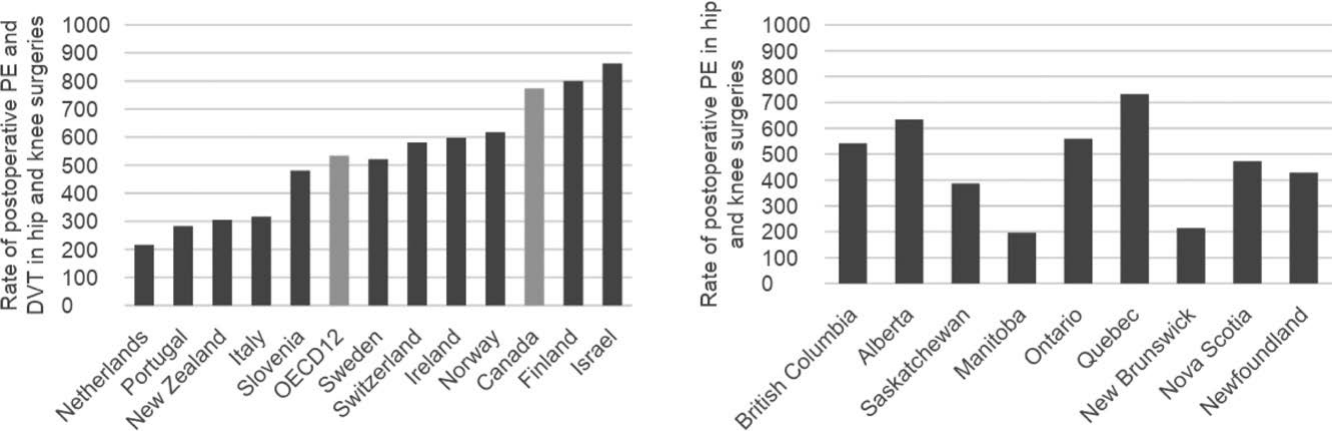
Comparing rates of postoperative pulmonary embolism and deep vein thrombosis in hip and knee surgeries; linked data from OECD and CIHI.^26,44^

The National Patient Safety Consortium defined eight consensus-derived Canadian Surgical Safety Indicators in 2016: wrong surgery (body part/procedure/patient), retained foreign body, surgical site infections, postoperative sepsis, postoperative deep vein thrombosis, postoperative pulmonary embolism, unplanned reoperation, surgical mortality, and patient-reported outcomes (eg, drug reaction, wound problems, postoperative pain, urinary problems).^25^ Many of these measures align with metrics established by CIHI used to compare health system outcomes within Canadian provinces and between Canada and other countries in the Organisation for Economic Co-operation and Development (OECD). These data identify critical and potentially actionable areas of national deficiency in surgical safety. For example, the rate of foreign body retention in Canada has increased by over 14% since 2012, which is more than double the average rate of 11 other countries reporting to the OECD (Figure 1; Canadian average: 9.8 per 100,000 hospital discharges vs OECD average: 4.0).^26^

The variation in the reporting methodology of adverse events leads to difficulty in comparisons and benchmarking even when there is agreement on specific metrics. Rates of preventable adverse events gleaned from administrative database studies are significantly lower than those found in the other studies, which use more comprehensive methods (eg, retrospective chart review, clinical surveillance). Clinical chart review, as used in the Canadian Adverse Events Study,^1^ is often regarded as the gold standard for identifying patient safety events, but it is imperfect, as the methodology is expensive, time-consuming, and requires regular data contribution and review to observe trends.^27–29^ Conversely, the CIHI study equips an administrative database methodology using the Discharge Abstract Database, a repository containing all discharge data from acute care hospitals in Canada. Acute care facilities across Canada, except in Quebec, are required to report data directly to CIHI, but the data obtained are not highly detailed.^7^ Data used by CIHI are prone to the errors in coding as well as the lack of detail inherent in administrative data collection. For example, CIHI rates do not account for all instances of harm, but are based on one or multiple instances of harm per patient.^6,7^ In addition, CIHI gathers surgical data from traditional hospital settings but does not collect information on the operations being performed in independent surgical facilities.^30–33^ Thus, national estimates of surgical harm frequently do not include such centers.

## PUBLIC–PRIVATE PARTNERSHIPS TO INCREASE ACCESS TO SURGICAL CARE

Diminished access to surgery and prolonged surgical wait times pose a safety risk for Canadian patients.^33,34^ Improving timely access to surgery is a priority of numerous provincial and national healthcare bodies (eg, health authorities and Ministries of Health) but simple solutions are scarce. Central intake models to maximize existing health system capacity provide a solution that is often relatively easily implemented and prioritizes access over physician autonomy.^35^ Public–private partnerships have been identified as a potential solution to expand surgical capacity. However, health systems must weigh the potential access benefits offered by these partnerships with risks. Partnership models risk harming public hospitals by siphoning off resources required for complex and high-acuity care and can also lead to increases in overall costs as well as a loss of public control and accountability. If these models are delivered without careful consideration, they run the risk of worsening healthcare disparities and potentially impacting the quality-of-care delivery.^36^

## STANDARDIZATION OF CARE AND IMPROVING SAFETY CULTURE

Some surgical safety solutions identified as the most critical by Canadian healthcare organizations also pose the most significant challenges for implementation and spread. These include standardizing safety procedures and practices, integrating the patient voice in quality and safety, and creating a culture of safety. The Canadian Medical Protective Agency released a 10-year review of medicolegal data in Canada, which found that the majority of patient harm was caused by deviation from hospital policy or procedure, clinical judgment issues, team-patient communication breakdown, lack of team coordination for the surgical safety checklist, or distractions.^37^ All of these could potentially be prevented by standardization, enhancing safety culture, and prioritizing the patient voice. Implementation of these approaches require that health systems move beyond traditional clinical outcome measures as a metric of success and capture patient experiences and measures of surgical culture. These solutions often require complex multimodal interventions that are best tailored and implemented at the local level. Scalable solutions include Enhanced Recovery programs.

## DISCUSSION

Within the Canadian health system, there are varied healthcare priorities, benchmarks, and methodologies to measure progress in surgical safety. There is no single metric to explain or measure the full extent of safety issues facing Canadians undergoing surgery. For Canadian health systems to leverage knowledge and address deficits, healthcare priorities must be aligned with reliable and measurable benchmarks. However, as has been noted by clinician leaders, data alone will not drive quality improvement.^13^ A disconnect exists between the priorities espoused by Canadian organizations and the benchmarks that our national databases collect (Table 5). Despite the value of the CIHI database, the surgical priorities outlined by Canadian healthcare bodies and organizations are not well reflected in the recorded data, they do not capture many patient-important outcomes, nor do they capture the most common adverse events occurring in Canada. While a ‘never event’, for example, retention of a foreign body, is easier to both measure and track, a more common adverse event such as postoperative delirium is less amenable to measurement and tracking. It is difficult to identify trends if standardized definitions cannot be agreed upon.

**TABLE 5.** Comparison of the National Benchmarks of Surgical Safety, Canadian Priorities, and Common Adverse Events (Accounting for Over 50% of All Harmful Events)

National Benchmarks^57^	Identified Canadian Priorities	Common Adverse Events^6^
Retained foreign body Postoperative PE in hip and knee surgery Postoperative sepsis in abdominal surgery Surgical readmissions Hospital deaths following major surgery	Preventable harm Never events Surgical site infections Safe medication delivery Perioperative fluid management and blood conservation	Electrolyte and fluid imbalance Urinary tract infections Delirium Anemia—hemorrhage Pneumonia Surgical site infections

Almost 20 years ago, the Canadian Adverse Events Study stated “there are few Canadian data on [adverse events] in hospital patients” (p. 1678),^1^ this remains true to this day, especially regarding surgical adverse events. Currently, only British Columbia, Manitoba, Nova Scotia, Ontario, Quebec, and Saskatchewan, publicly report trends in patient harm events.^36,38–42^ This lack of consistent, reliable tracking metrics limits the ability to identify worrying trends or successful initiatives that are improving surgical safety.

Despite national and international endeavors aimed at improving safety, the overall rate of adverse events in surgery is increasing.^6^ A renewed national strategy for surgical safety is needed. Such a strategy requires co-operation and collaboration at the national, provincial, and organizational level in a manner that incorporates national safety priorities. This national strategy must strike a balance between focusing on patient harm and what is needed to create safer care, as the absence of harm does not necessarily mean that care is safe.^43^ Solutions must address the perspectives of both those providing and receiving care.^43^ Understanding and unifying these priorities will create a foundation upon which national benchmarks can be determined and standardized reporting can occur. If we want to curb the rise of adverse events and safety incidents in Canada, it is imperative we reassess our safety strategy.

## ACKNOWLEDGMENTS

All the authors contributed to the development, writing, and editing of the manuscript.

## Supplementary Material
