# Supplementary material for: Healthcare Priorities for Surgical Care in Canada
Source: Ann Surg Open. 2025 Apr 4;6(2):e561. doi: 10.1097/AS9.0000000000000561 (PMC12185075; doi:10.1097/AS9.0000000000000561)
Supplement: Supplementary file 1 [file as9-6-e561-s001.pdf]

Supplementary Material

Supplementary Material 1: Surgical safety priorities identified by Canadian healthcare organizations

Surgical priorities	Organizations
1. Addressing surgical backlogs and efficiencies	n=25; Alberta Health Services, Alberta Ministry of Health, BC Ministry of Health, BC Provincial Health Services Authority, Canadian Institute for Health Information, Canadian Medical Association, Canadian Nurses Association, Health Canada, Health PEI, Health Quality Ontario, Horizon Health Network (NB), Manitoba Health, New Brunswick Department of Health, Newfoundland & Labrador Department of Health and Community Services, NL Health Services, Nova Scotia Department of Health and Wellness, Nova Scotia Health, Ontario Ministry of Health, PEI Department of Health and Wellness, Quebec Ministry of Health and Social Services, Saskatchewan Health Authority, Saskatchewan Ministry of Health, Shared Health Manitoba, Vitalité Health Network (NB), Yukon Health and Social Services
2. Improving patient safety and quality of care	n=17; Accreditation Canada, Alberta Health Services, BC Provincial Health Services Authority, Best Practices in Surgery, Canadian Institute for Health Information, Health Canada, Healthcare Excellence Canada, Health PEI, Health Quality Council of Alberta, Health Quality Ontario, Healthcare Insurance Reciprocal of Canada, Horizon Health Network (NB), NL Health Services, Northwest Territories Department of Health and Social Services, Nova Scotia Health, Operating Room Nurses Association of Canada, Patients for Patient Safety Canada
3. Improving patient outcomes	n=15; BC Patient Safety & Quality Council, Best Practices in Surgery, Canadian Institute for Health Information, Canadian Medical Protective Association, Healthcare Excellence Canada, Enhanced Recovery Canada, Health PEI, Health Quality Ontario, Healthcare Insurance Reciprocal of Canada, Horizon Health Network (NB), Manitoba Health, NL Health Services, Northwest Territories Department of Health and Social Services, Nova Scotia Health, Vitalité Health Network (NB)
4. Improving care coordination and surgical pathways (e.g., handovers, transitions)	n=12; Alberta Health Services, Best Practices in Surgery, Canadian Medical Protective Association, Health PEI, Healthcare Excellence Canada, NL Health Services, Horizon Health Network (NB), New Brunswick Department of Health, Nova Scotia Department of Health and Wellness, Nova Scotia Health, Shared Health Manitoba, Vitalité Health Network (NB)
5. Establishing perioperative service standards and best practices	n=12; Accreditation Canada, BC Centre for Disease Control, Best Practices in Surgery, Canadian Anesthesiologists' Society, Canadian Medical Protective Association, Canadian Society of Respiratory Therapists, Enhanced Recovery Canada, Horizon Health Network (NB), New Brunswick Department of Health, Nova Scotia Department of Health and Wellness, Nova Scotia Health, Royal College of Physicians and Surgeons of Canada
6. Improving surgical culture, teamwork, and communication	n=8; Accreditation Canada, Alberta Health Services, BC Patient Safety & Quality Council, Canadian Anesthesiologists' Society, Canadian Medical Protective Association, Healthcare Excellence Canada, Health Quality Ontario, Northwest Territories Department of Health and Social Services

7. Reducing preventable harm and never events (e.g., foreign body retention, wrong-side/patient/procedure surgery)	n=7; Canadian Institute for Health Information, Canadian Medical Protective Association, Healthcare Excellence Canada, Health Quality Ontario, Healthcare Insurance Reciprocal of Canada, Nova Scotia Department of Health and Wellness, Patients for Patient Safety Canada
8. Reducing surgical site infections	n=5; Best Practices in Surgery, Canadian Nosocomial Infection Surveillance Program, Health Quality Ontario, Infection Protection & Control Canada, Ontario Ministry of Health

Supplementary Material 2: Priority healthcare issues identified by Canadian healthcare organizations

Healthcare priorities	Organizations
1. Addressing access to care and wait times	n=25; Alberta Health Services, BC Centre for Disease Control, BC Ministry of Health, BC Provincial Health Services Authority, Canadian Anesthesiologists' Society, Canadian Institute for Health Information, Canadian Medical Association, Canadian Nurses Association, Canadian Society of Respiratory Therapists, Health Canada, Health Quality Ontario, Healthcare Excellence Canada, Manitoba Health, New Brunswick Department of Health, Nova Scotia Department of Health and Wellness, Nova Scotia Health, Quebec Ministry of Health and Social Services, Saskatchewan Ministry of Health, Shared Health Manitoba, Yukon Health and Social Services
2. Mental health and addictions	n=25; Alberta Health Services, Alberta Ministry of Health, BC Centre for Disease Control, BC Ministry of Health, Canadian Institute for Health Information, Canadian Medical Association, Canadian Nurses Association, Health Canada, Health PEI, Health Quality Ontario, Horizon Health Network (NB), New Brunswick Department of Health, Newfoundland & Labrador Department of Health and Community Services, NL Health Services, Northwest Territories Department of Health and Social Services, Nova Scotia Department of Health and Wellness, Nova Scotia Health, Ontario Ministry of Health, PEI Department of Health and Wellness, Quebec Ministry of Health and Social Services, Saskatchewan Health Authority, Saskatchewan Health Quality Council, Saskatchewan Ministry of Health, Vitalité Health Network (NB), Yukon Health and Social Services
3. Long-term care	n=16; Alberta Ministry of Health, BC Ministry of Health, Health Canada, Health PEI, Healthcare Excellence Canada, Infection Protection and Control Canada, Manitoba Health, New Brunswick Health Council, New Brunswick Department of Health, Newfoundland & Labrador Department of Health and Community Services, NL Health Services, Nova Scotia Health, PEI Department of Health and Wellness, Quebec Ministry of Health and Social Services, Saskatchewan Ministry of Health, Yukon Health and Social Services
4. Opioid crisis	n=10; BC Ministry of Health, BC Provincial Health Services Authority, Best Practices in Surgery, Canadian Society of Hospital Pharmacists, Health Canada, Newfoundland & Labrador Department of Health and Community Services,

	Northwest Territories Department of Health and Social Services, Nova Scotia Health, Ontario Ministry of Health, Yukon Health and Social Services
5. Antimicrobial resistance	n=7; Alberta Health Services, BC Centre for Disease Control, Canadian Nosocomial Infection Surveillance Program, Canadian Nurses Association, Health Canada, Infection Protection and Control Canada, Patients for Patient Safety Canada
